# Results from the Survey of Antibiotic Resistance (SOAR) 2018–21 in India: data based on CLSI, EUCAST (dose-specific) and pharmacokinetic/pharmacodynamic (PK/PD) breakpoints

**DOI:** 10.1093/jac/dkaf285

**Published:** 2025-11-24

**Authors:** Didem Torumkuney, Balaji Veeraraghavan, Niranjan Patil, Mary Dias, Geeti Maheshwari, Bhaskar Narayan Chaudhuri, Ujjwayini Ray, Stephen Hawser, Subhashri Kundu, Anand Manoharan

**Affiliations:** Infectious Diseases Research Unit, GSK, London, UK; Department of Clinical Microbiology, Christian Medical College, Vellore, India; Microbiology and Infectious Molecular Biology, Metropolis Healthcare Private Limited, Vidyavihar West, Mumbai, India; Department of Microbiology and Division of Infectious Diseases, St John's Medical College Hospital, Bangalore, India; Department of Microbiology and Molecular Sciences, Toprani Advanced Lab Systems, Vadodara, India; Microbiology Research Center LTD, Peerless Hospital, Kolkata, India; Consultant, Apollo Multispecialty Hospitals, Kolkata, India; Global Affairs, IHMA Europe Sàrl, Monthey, Switzerland; Infectious Diseases - Antibiotic Resistance, GSK, Singapore, Republic of Singapore; Infectious Diseases Medical & Scientific Affairs, GSK, Mumbai, India

## Abstract

**Objectives:**

Antibiotic susceptibility determination of community-acquired respiratory tract infection (CA-RTI) isolates of *Streptococcus pneumoniae* and *Haemophilus influenzae* were collected from India (2018–21).

**Methods:**

MICs were determined by CLSI broth microdilution; susceptibility data were interpreted using CLSI, EUCAST and pharmacokinetic/pharmacodynamic (PK/PD) breakpoints.

**Results:**

*S. pneumoniae* (*n* = 177) and *H. influenzae* (*n* = 171) isolates were collected from four hospital laboratories and two private laboratories in India. Only 41.2% pneumococci were penicillin susceptible by CLSI oral/EUCAST low-dose breakpoints, but 94.4% were susceptible by EUCAST high-dose/CLSI IV breakpoints. Good activity (≥89.8%, CLSI or PK/PD breakpoints) was observed for amoxicillin, amoxicillin/clavulanic acid, cefotaxime, ceftriaxone, levofloxacin, and moxifloxacin. Cefdinir and second-generation cephalosporins were less active (27.7%–64.4%). Tetracyclines, macrolides and trimethoprim/sulfamethoxazole showed poor activity (18.6%–31.1%). EUCAST breakpoints indicated >90% susceptibility to high-dose ceftriaxone and penicillin, moxifloxacin and high-dose levofloxacin. Lower susceptibility to other cephalosporins and aminopenicillins was observed with EUCAST versus CLSI or PK/PD breakpoints. Most *H. influenzae* isolates (91.8%) were β-lactamase negative; 13 and 5 were β-lactamase-negative ampicillin-resistant following EUCAST and CLSI criteria, respectively. Antibiotic susceptibility was ≥84.8% (CLSI) for all antibiotics except trimethoprim/sulfamethoxazole (23.4%). Susceptibility by EUCAST was similar, except for cefuroxime (oral) with no susceptible isolates versus 95.3% by CLSI and ≤29.8% versus ≥85.4% for fluoroquinolones.

**Conclusions:**

Some therapeutic options against *S. pneumoniae* and *H. influenzae* from CA-RTI in India remain, but only ceftriaxone covers both bacterial species using both guidelines. Continued surveillance of antibiotic susceptibility is important to monitor changes and trends in susceptibilities and for guiding empiric therapy of CA-RTIs.

## Introduction

Community-acquired respiratory tract infections (CA-RTIs) are an important world health problem. If treated incorrectly, or in patients with comorbidities, CA-RTIs can result in hospitalization, with a third of patients dying from pneumonia within 12 months of being discharged from hospital.^1^ However, comorbidities, age and other risk factors might have contributed to the mortality rate.^1^ Treatment of CA-RTIs is reliant on empirical antibiotic therapy through the use of national and international guidelines.^2^ Studies have shown a high level of inappropriate antibiotic use in primary and tertiary care centres in India, including incomplete prescriptions, fixed-dose combinations, unapproved formulations and self-medication,^3–6^ leading to increasing antibiotic resistance.^7^ In 2015, India was shown to be the highest consumer of antibiotics amongst low- and middle-income countries.^8^ In light of this, the Indian Government initiated a national action plan in 2017 in response to the WHO’s global action plan to combat antimicrobial resistance.^9^

*Streptococcus pneumoniae* and *Haemophilus influenzae* are the major bacteria associated with CA-RTIs.^10,11^ Both pathogens have shown increasing resistance to first-line antibiotics such as penicillin and ampicillin.^12,13^ As rates of resistance vary over time and from country to country, up-to-date surveillance data are essential to guide local antibiotic policies.^14^

The Survey of Antibiotic Resistance (SOAR), an international antibiotic resistance surveillance study, focuses on key respiratory pathogens that cause community-acquired infections and has been running since 2002 in the Middle East, Africa, Latin America, Asia-Pacific, Europe and the Commonwealth of Independent States countries.^15^ For this study, recent SOAR data from hospitals in India have been analysed to provide a picture of the current state of antibiotic susceptibility of *S. pneumoniae* and *H. influenzae* associated with CA-RTIs.

## Materials and methods

### Ethics

SOAR studies are not human subject studies. During the study, only microorganisms were examined.

### Collaborating centres

Isolates were collected between 2018 and 2021 from four hospitals (St John’s Medical College Hospital, Bangalore; Peerless Hospital, Kolkata; Apollo Gleneagles Hospitals, Kolkata; and Christian Medical College, Vellore) and two laboratories (Toprani Advanced Lab Systems, Vadodara; and Metropolis Healthcare Ltd, Kurla) located throughout India.

### Clinical isolates

Isolates of *H. influenzae* and *S. pneumoniae* from CA-RTIs (isolated within 48 h of hospitalization) were sent to a central laboratory (IHMA Europe, Monthey, Switzerland), where they were sub-cultured and re-identified. *H. influenzae* were re-identified by MALDI-TOF MS methodology and *S. pneumoniae* identity was confirmed by optochin susceptibility and bile solubility tests. β-lactamase production was determined for each *H. influenzae* isolate by a chromogenic cephalosporin (nitrocefin) disc method. Duplicate isolates from the same patient were not accepted.

### Antimicrobial susceptibility testing

Isolates were evaluated for antibiotic susceptibility using broth microdilution methodology recommended by CLSI.^16^ Amoxicillin, amoxicillin/clavulanic acid (2:1 ratio as per CLSI guidelines^16,17^), amoxicillin/clavulanic acid (fixed clavulanic acid at 2 mg/L as per EUCAST guidelines^18^), azithromycin, cefaclor, cefdinir, cefixime, cefotaxime, cefpodoxime, ceftibuten, ceftriaxone, cefuroxime, clarithromycin, levofloxacin, moxifloxacin and trimethoprim/sulfamethoxazole (1:19 ratio) were tested against both respiratory pathogens. In addition, doxycycline, erythromycin and penicillin were tested against *S. pneumoniae* only, and ampicillin was tested against *H. influenzae* only. Susceptibility to the study drugs was calculated based on CLSI, EUCAST (dose-specific) and pharmacokinetic/pharmacodynamic (PK/PD) breakpoints.^17–19^ These breakpoints are shown in Tables 1–3. To fully assess antibiotics where high-dose therapies are available, susceptibility using EUCAST criteria was also calculated by using the susceptible, increased exposure breakpoints (former intermediate category) as well as dose-dependent PK/PD breakpoints.^18^

**Table 1.** CLSI MIC breakpoints (mg/L) used for *S. pneumoniae* and *H. influenzae* isolates

	*S. pneumoniae*			*H. influenzae*		
Antimicrobial	S	I	R	S	I	R
Amoxicillin	≤2	4	≥8	—	—	—
Amoxicillin/clavulanic acid (2:1)^a^	≤2	4	≥8	≤2	4	≥8
Ampicillin	NT	NT	NT	≤1	2	≥4
Azithromycin	≤0.5	1	≥2	≤4	—	—
Cefaclor	≤1	2	≥4	≤8	16	≥32
Cefdinir	≤0.5	1	≥2	≤1	—	—
Cefixime	—	—	—	≤1	—	—
Cefotaxime (non-meningitis)	≤1	2	≥4	≤2	—	—
Cefpodoxime	≤0.5	1	≥2	≤2	—	—
Ceftibuten	—	—	—	≤2	—	—
Ceftriaxone (non-meningitis)	≤1	2	≥4	≤2	—	—
Cefuroxime^b^	≤1	2	≥4	≤4	8	≥16
Clarithromycin	≤0.25	0.5	≥1	≤8	16	≥32
Doxycycline	≤0.25	0.5	≥1	NT	NT	NT
Erythromycin	≤0.25	0.5	≥1	NT	NT	NT
Levofloxacin	≤2	4	≥8	≤2	—	—
Moxifloxacin	≤1	2	≥4	≤1	—	—
Penicillin (2.4 g 2 MU × 4–6 IV)	≤2	4	≥8	NT	NT	NT
Penicillin (oral)	≤0.06	0.12–1	≥2	NT	NT	NT
Tetracycline	≤1	2	≥4	≤2	4	≥8
Trimethoprim/sulfamethoxazole^c^	≤0.5	1–2	≥4	≤0.5	1–2	≥4

—, not applicable; I, intermediate; NT, not tested; R, resistant; S, susceptible.

^a^Amoxicillin/clavulanic acid was tested at a 2:1 amoxicillin to clavulanic acid ratio; breakpoints are expressed as the amoxicillin component.

^b^Breakpoints used are for cefuroxime axetil (oral).

^c^Trimethoprim/sulfamethoxazole was tested at a 1:19 trimethoprim to sulfamethoxazole ratio; breakpoints are expressed as the trimethoprim component.

**Table 2.** EUCAST (dose-specific) MIC breakpoints (mg/L) used for *S. pneumoniae* and *H. influenzae* isolates

	*S. pneumoniae*		*H. influenzae*	
Antimicrobial^a^	S	R	S	R
Amoxicillin (0.5 g × 3 oral)	≤0.5	>1	≤0.001	>2
Amoxicillin (0.75–1 g × 3 oral)	≤1	>1	≤2	>2
Amoxicillin/clavulanic acid (0.5 g/0.125 g × 3 oral)^b^	≤0.5	>1	≤0.001	>2
Amoxicillin/clavulanic acid (0.875 g/0.125 g × 3 oral)^b^	≤1	>1	≤2	>2
Ampicillin (2 g × 3 IV)	NT	NT	≤1	>1
Ampicillin (2 g × 4 IV)	NT	NT	≤1	>1
Azithromycin	≤0.25	>0.5	—	—
Cefaclor	≤0.001	>0.5	—	—
Cefdinir	—	—	—	—
Cefixime	—	—	≤0.12	>0.12
Cefotaxime	≤0.5	>2	≤0.12	>0.12
Cefpodoxime	≤0.25	>0.5	≤0.25	>0.25
Ceftibuten	—	—	≤1	>1
Ceftriaxone (1 g × 1 IV)	≤0.5	>2	≤0.12	>0.12
Ceftriaxone (2 g × 2 IV)	≤2	>2	≤0.12	>0.12
Cefuroxime^c^	≤0.25	>0.5	≤0.001	>1
Clarithromycin (0.25 g × 2 oral)	≤0.25	>0.5	—	—
Clarithromycin (0.5 g × 2 oral)	≤0.5	>0.5	—	—
Doxycycline	≤1	>2	NT	NT
Erythromycin (0.5 g × 2–4 oral or 0.5 g × 2–4 IV)	≤0.25	>0.5	NT	NT
Erythromycin (1 g × 4 oral or 1 g × 4 IV)	≤0.5	>0.5	NT	NT
Levofloxacin (0.5 g × 2 oral or 0.4 g × 2 IV)	≤0.001	>2	≤0.06	>0.06
Levofloxacin (0.75 g × 2 oral or 0.4 g × 3 IV)	≤2	>2	≤0.06	>0.06
Moxifloxacin	≤0.5	>0.5	≤0.12	>0.12
Penicillin (0.6 g 1 MU × 4 IV)	≤0.06	>2	NT	NT
Penicillin (2.4 g 2 MU × 4–6 IV)	≤2	>2	NT	NT
Tetracycline	≤1	>2	≤2	>2
Trimethoprim/sulfamethoxazole (0.16 g/0.8 g × 2 oral or IV)^d^	≤1	>2	≤0.5	>1
Trimethoprim/sulfamethoxazole (0.24 g/1.2 g × 2 oral or IV)^d^	≤2	>2	≤1	>1

—, not applicable; NT, not tested; R, resistant; S, susceptible.

^a^Where available, susceptibility was assessed using EUCAST higher-dosage breakpoints.

^b^Amoxicillin/clavulanic acid was tested at a fixed concentration of 2 mg/L; breakpoints are expressed as the amoxicillin component.

^c^Breakpoints used are for cefuroxime axetil (oral).

^d^Trimethoprim/sulfamethoxazole was tested at a 1:19 trimethoprim to sulfamethoxazole ratio; breakpoints are expressed as the trimethoprim component.

**Table 3.** PK/PD MIC breakpoints (mg/L) used for *S. pneumoniae* and *H. influenzae* isolates

	*S. pneumoniae* and *H. influenzae*
Antimicrobial	S only
Amoxicillin (1.5 g/day)^a^	≤2
Amoxicillin (4 g/day)^b^	≤4
Amoxicillin/clavulanic acid^a^ (1.75 g/0.25 g/day adults; 45 mg/6.4 mg/kg/day children)	≤2
Amoxicillin/clavulanic acid^b^ (4 g/0.25 g/day adults; 90 mg/6.4 mg/kg/day children)	≤4
Ampicillin	—
Penicillin	—
Cefaclor	≤0.5
Cefdinir	≤0.25
Cefditoren	—
Cefixime	≤1
Cefpodoxime	≤0.5
Ceftriaxone	≤1
Cefuroxime^c^	≤1
Azithromycin	≤0.12
Clarithromycin	≤0.25
Erythromycin	≤0.25
Levofloxacin	≤2
Moxifloxacin	≤1
Trimethoprim/sulfamethoxazole^d^	≤0.5

—, not applicable; PK/PD, pharmacokinetic/pharmacodynamic; S, susceptible.

^a^Amoxicillin/clavulanic acid for low dose in adults/children.

^b^Amoxicillin/clavulanic acid for high dose in adults/children.

^c^Breakpoints used are for cefuroxime axetil (oral).

^d^Trimethoprim/sulfamethoxazole was tested at a 1:19 trimethoprim to sulfamethoxazole ratio; breakpoints are expressed as the trimethoprim component.

### Quality control and data analysis

Quality control strains *S. pneumoniae* ATCC 49619, *H. influenzae* ATCC 49247, *H. influenzae* ATCC 49766 and *E. coli* ATCC 32518 were included on each day of testing. Results of susceptibility testing were only accepted if the results of the quality control strains were within the published acceptable range. Differences in susceptibility (using CLSI criteria only) across penicillin-susceptible (PSSP) isolates (*S. pneumoniae* only) were assessed for statistical significance with Fisher’s exact test using XLSTAT version 2023.1.1.1399. A *P*-value <0.05 was considered statistically significant. A similar statistical analysis was made to compare the susceptibility of isolates from 2018 to 2021 with India SOAR data from 2012 to 2014 (using CLSI criteria).^15,20^ The amoxicillin/clavulanate breakpoints have been lowered by CLSI since the 2012–14 study; therefore, to make a fair comparison, susceptibility from 2012 to 2014 was adjusted to conform with current CLSI breakpoints.

## Results

### *S. pneumoniae* isolates

A total of 177 *S. pneumoniae* isolates were collected in India between 2018 and 2021. Most isolates came from sputum (*n* = 142, 80.2%) with the remainder from blood (*n* = 19, 10.7%), bronchoalveolar lavage (*n* = 7, 4.0%), middle ear (*n* = 1, 0.6%) and unidentified specimens (*n* = 8, 4.5%). The majority of isolates (*n* = 122, 68.9%) came from adolescent and adult patients (aged 13–64 years), 35 (19.8%) isolates were from elderly patients (aged ≥65 years) and 20 (11.3%) isolates from paediatric patients (aged ≤12 years).

**Figure 1.**
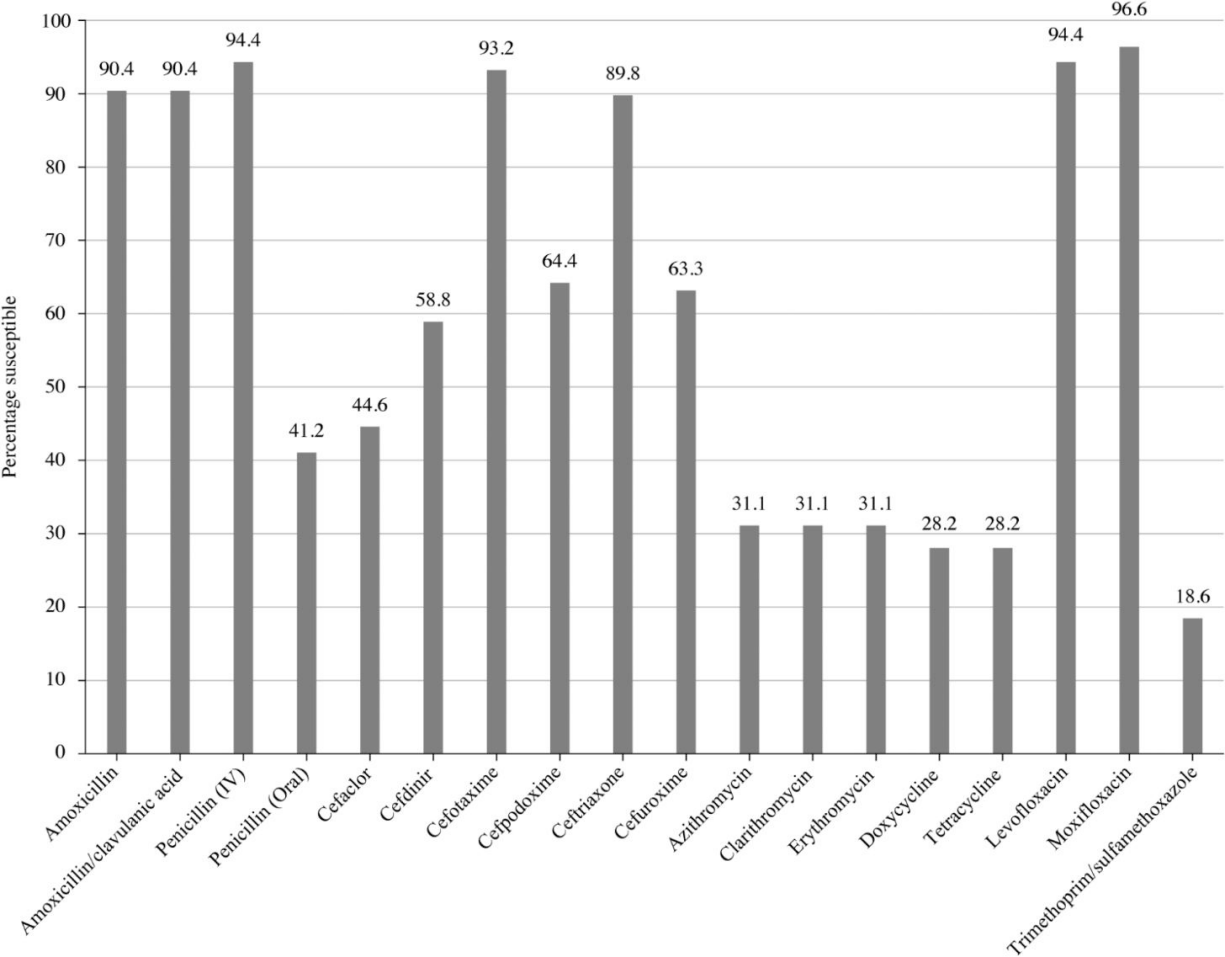
Antibiotic susceptibility rates of *S. pneumoniae* isolates (*n* = 177) from India based on CLSI breakpoints.

**Figure 2.**
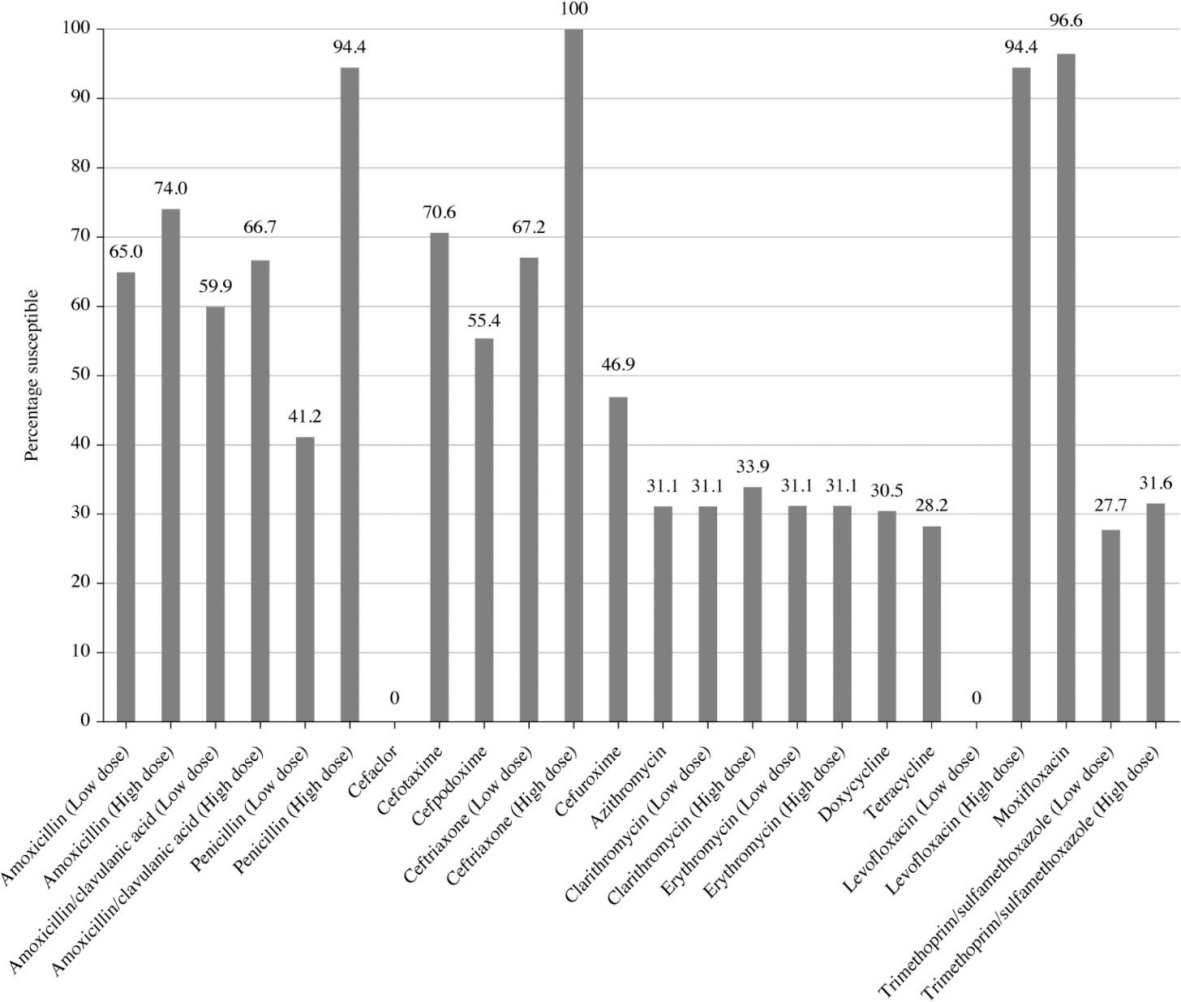
Antibiotic susceptibility rates of *S. pneumoniae* isolates (*n* = 177) from India based on EUCAST (dose-specific) breakpoints.

Summary MIC, susceptibility and MIC distribution data for all 177 *S. pneumoniae* isolates are shown in Tables 4–6 and S1 (available as Supplementary data at *JAC* Online) and Figures 1 and 2.

**Table 4.** MIC and susceptibility data for *S. pneumoniae* isolates (*n* = 177) from India using CLSI breakpoints

	MIC (mg/L)			CLSI susceptibility		
Antimicrobial	Range	50%	90%	S (%)	I (%)	R (%)
Amoxicillin	0.015 to 8	0.12	2	90.4	4.0	5.6
Amoxicillin/clavulanic acid (2:1)	≤0.008 to 8	0.12	2	90.4	4.0	5.6
Penicillin (2.4 g 2 MU × 4–6 IV)	≤0.008 to 4	0.25	2	94.4	5.6	0
Penicillin (oral)	≤0.008 to 4	0.25	2	41.2	37.9	20.9
Cefaclor	0.25 to >4	2	>4	44.6	11.3	44.1
Cefdinir	0.03 to >8	0.5	8	58.8	5.6	35.6
Cefixime	≤0.25 to >16	2	>16	—	—	—
Cefotaxime	≤0.008 to 2	0.25	1	93.2	6.8	0
Cefpodoxime	≤0.015 to >4	0.25	4	64.4	5.6	29.9
Ceftibuten	2 to >16	>16	>16	—	—	—
Ceftriaxone	0.015 to 2	0.25	2	89.8	10.2	0
Cefuroxime	≤0.008 to 8	0.5	8	63.3	4.0	32.8
Azithromycin	≤0.015 to >16	8	>16	31.1	0	68.9
Clarithromycin	≤0.015 to >16	2	>16	31.1	2.8	66.1
Erythromycin	≤0.015 to >16	4	>16	31.1	0	68.9
Doxycycline	0.03 to >4	4	>4	28.2	0.6	71.2
Tetracycline	0.12 to >4	>4	>4	28.2	0.6	71.2
Levofloxacin	0.25 to >8	1	1	94.4	0.6	5.1
Moxifloxacin	≤0.03 to 2	0.12	0.12	96.6	3.4	0
Trimethoprim/sulfamethoxazole	0.12 to >8	4	8	18.6	13.0	68.4

—, not applicable; I, intermediate; R, resistant; S, susceptible.

**Table 5.** MIC and susceptibility data for *S. pneumoniae* isolates (*n* = 177) from India using EUCAST (dose-specific) breakpoints

	MIC (mg/L)			EUCAST susceptibility		
Antimicrobial	Range	50%	90%	S (%)	I (%)	R (%)
Amoxicillin (0.5 g × 3 oral)	0.015 to 8	0.12	2	65.0	9.0	26.0
Amoxicillin (0.75–1 g × 3 oral)	0.015 to 8	0.12	2	74.0	—	26.0
Amoxicillin/clavulanic acid (0.5 g/0.125 g × 3 oral)	≤0.008 to >8	0.25	8	59.9	6.8	33.3
Amoxicillin/clavulanic acid (0.875 g/0.125 g × 3 oral)	≤0.008 to >8	0.25	8	66.7	—	33.3
Penicillin (0.6 g 1 MU × 4 IV)	≤0.008 to 4	0.25	2	41.2	53.1	5.6
Penicillin (2.4 g 2 MU × 4–6 IV)	≤0.008 to 4	0.25	2	94.4	—	5.6
Cefaclor	0.25 to >4	2	>4	0	27.7	72.3
Cefdinir	0.03 to >8	0.5	8	—	—	—
Cefixime	≤0.25 to >16	2	>16	—	—	—
Cefotaxime	≤0.008 to 2	0.25	1	70.6	29.4	0
Cefpodoxime	≤0.015 to >4	0.25	4	55.4	9.0	35.6
Ceftibuten	2 to >16	>16	>16	—	—	—
Ceftriaxone (1 g × 1 IV)	0.015 to 2	0.25	2	67.2	32.8	0
Ceftriaxone (2 g × 2 IV)	0.015 to 2	0.25	2	100	—	0
Cefuroxime	≤0.008 to 8	0.5	8	46.9	16.4	36.7
Azithromycin	≤0.015 to >16	8	>16	31.1	0	68.9
Clarithromycin (0.25 g × 2 oral)	≤0.015 to >16	2	>16	31.1	2.8	66.1
Clarithromycin (0.5 g × 2 oral)	≤0.015 to >16	2	>16	33.9	—	66.1
Erythromycin (0.5 g × 2–4 oral or 0.5 g × 2–4 IV)	≤0.015 to >16	4	>16	31.1	0	68.9
Erythromycin (1 g × 4 oral or 1 g × 4 IV)	≤0.015 to >16	4	>16	31.1	—	68.9
Doxycycline	0.03 to >4	4	>4	30.5	5.6	63.8
Tetracycline	0.12 to >4	—	—	28.2	0.6	71.2
Levofloxacin (0.5 g × 2 oral or 0.4 g × 2 IV)	0.25 to >8	1	1	0	94.4	5.6
Levofloxacin (0.75 g × 2 oral or 0.4 g × 3 IV)	0.25 to >8	1	1	94.4	—	5.6
Moxifloxacin	≤0.03 to 2	0.12	0.12	96.6	—	3.4
Trimethoprim/sulfamethoxazole (0.16 g/0.8 g × 2 oral or IV)	0.12 to >8	4	8	27.7	4.0	68.4
Trimethoprim/sulfamethoxazole (0.24 g/1.2 g × 2 oral or IV)	0.12 to >8	4	8	31.6	—	68.4

—, not applicable; I, susceptible, increased exposure; R, resistant; S, susceptible.

**Table 6.** Summary MIC and susceptibility data for *S. pneumoniae* (*n* = 177) from India using PK/PD breakpoints

	MIC (mg/L)			PK/PD susceptibility
Antimicrobial	Range	50%	90%	S (%)
Amoxicillin (1.5 g/day)	0.015 to 8	0.12	2	90.4
Amoxicillin (4 g/day)	0.015 to 8	0.12	2	94.4
Amoxicillin/clavulanic acid (1.75 g/0.25 g/day adults; 45 mg/6.4 mg/kg/day children)	≤0.008 to 8	0.12	2	90.4
Amoxicillin/clavulanic acid (4 g/0.25 g/day adults; 90 mg/6.4 mg/kg/day children)	≤0.008 to 8	0.12	2	94.4
Penicillin	≤0.008 to 4	0.25	2	—
Cefaclor	0.25 to >4	2	>4	27.7
Cefdinir	0.03 to >8	0.5	8	48.6
Cefixime	≤0.25 to >16	2	>16	38.4
Cefotaxime	≤0.008 to 2	0.25	1	—
Cefpodoxime	≤0.015 to >4	0.25	4	64.4
Ceftibuten	2 to >16	>16	>16	—
Ceftriaxone	0.015 to 2	0.25	2	89.8
Cefuroxime	≤0.008 to 8	0.5	8	63.3
Azithromycin	≤0.015 to >16	8	>16	28.2
Clarithromycin	≤0.015 to >16	2	>16	31.1
Erythromycin	≤0.015 to >16	4	>16	31.1
Doxycycline	0.03 to >4	4	>4	28.2
Tetracycline	0.12 to >4	>4	>4	—
Levofloxacin	0.25 to >8	1	1	94.4
Moxifloxacin	≤0.03 to 2	0.12	0.12	96.6
Trimethoprim/sulfamethoxazole	0.12 to >8	4	8	18.6

—, not applicable; PK/PD, pharmacokinetic/pharmacodynamic; S, susceptible.

### *S. pneumoniae* susceptibility

The penicillin susceptibility of the pneumococci from India following CLSI oral or EUCAST low-dose IV breakpoints was 41.2%, but with EUCAST high-dose and CLSI IV breakpoints, susceptibility increased to 94.4%. Following CLSI breakpoints, amoxicillin, amoxicillin/clavulanic acid and the third-generation cephalosporins (ceftriaxone and cefotaxime) showed similar activity (89.8%–93.2% susceptible), but against the third-generation cephalosporin cefdinir, 58.8% were susceptible. The second-generation cephalosporins cefpodoxime, cefuroxime and cefaclor showed susceptibility of 64.4%, 63.3% and 44.6%, respectively. Similar activity was observed using PK/PD breakpoints for these agents, although high-dose amoxicillin or amoxicillin/clavulanic acid PK/PD breakpoints increased susceptibility to 94.4%. The use of EUCAST breakpoints, conversely, indicated lower susceptibility than with CLSI breakpoints to amoxicillin, amoxicillin/clavulanic acid and all cephalosporins except high-dose ceftriaxone. Only high-dose ceftriaxone and high-dose penicillin had >90% susceptibility using EUCAST breakpoints against pneumococci from India. Weak activity (18.6%–33.9% susceptibility) was observed for the macrolides (azithromycin, clarithromycin and erythromycin), tetracyclines (doxycycline and tetracycline) and trimethoprim/sulfamethoxazole by CLSI, EUCAST and PK/PD interpretive criteria. Moxifloxacin susceptibility was 96.6% following all three breakpoints, and, similarly, levofloxacin susceptibility was 94.4% by CLSI, EUCAST high-dose and PK/PD breakpoints (Tables 4–6 and Figures 1 and 2).

### Susceptibility of *S. pneumoniae* by penicillin resistance phenotype

**Figure 3.**
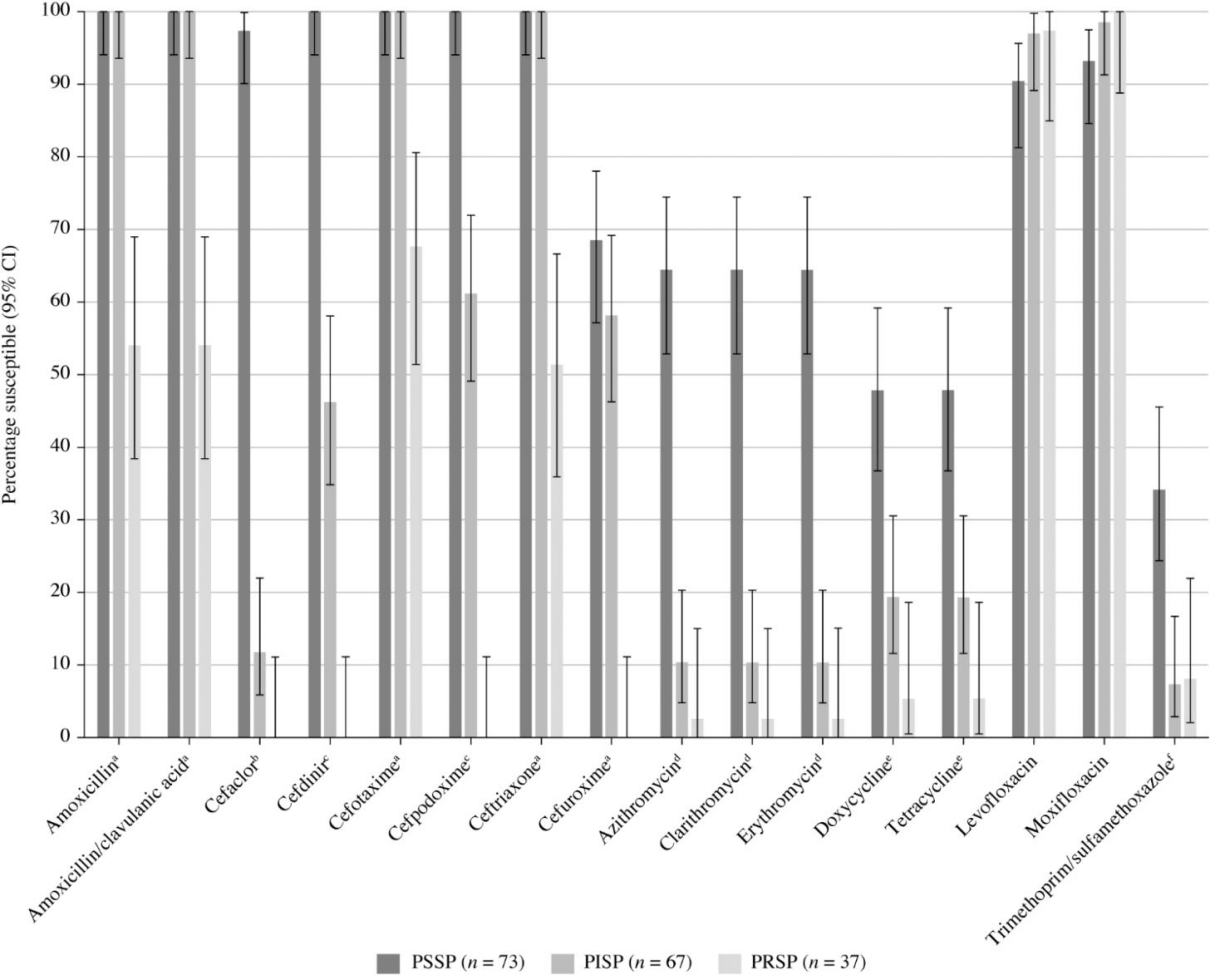
Susceptibility rates (with 95% CI) based on CLSI breakpoints for antibiotics against PSSP, PISP and PRSP from India. Penicillin susceptibility categories are based on oral penicillin CLSI breakpoints. ^a^Susceptibility was significantly higher among PSSP and PISP isolates than PRSP isolates (*P* < 0.0001). ^b^Susceptibility was significantly higher among PSSP than PRSP isolates (*P* < 0.0001), significantly higher among PISP than PRSP isolates (*P* = 0.006) and significantly higher among PSSP than PISP isolates (*P* < 0.0001). ^c^Susceptibility was significantly higher among PSSP and PISP isolates than PRSP isolates and significantly higher among PSSP than PISP isolates (*P* < 0.0001). ^d^Susceptibility was significantly higher among PSSP than PISP or PRSP isolates (*P* < 0.0001). ^e^Susceptibility was significantly higher among PSSP than PRSP isolates (*P* < 0.0001), significantly higher among PISP than PRSP isolates (*P* = 0.035) and significantly higher among PSSP than PISP isolates (*P* = 0.0006). ^f^Susceptibility was significantly higher among PSSP than PISP or PRSP isolates (*P* = 0.0001). PISP, penicillin-intermediate *S. pneumoniae*; PRSP, penicillin-resistant *S. pneumoniae*; PSSP, penicillin-susceptible *S. pneumoniae*.

Of the 177 *S. pneumoniae* isolates collected, 73 (41.2%) were PSSP, 67 (37.9%) were penicillin intermediate (PISP) and 37 (20.9%) were penicillin resistant (PRSP) according to CLSI oral breakpoints (Figure 3). The PSSP isolates were ≥90% susceptible to amoxicillin, amoxicillin/clavulanic acid, all cephalosporins (except cefuroxime) and fluoroquinolones. Conversely, susceptibility of PSSP to cefuroxime and macrolides was only 64.4%–68.5%, and susceptibility to tetracyclines and trimethoprim/sulfamethoxazole was 47.9% and 34.2%, respectively. PSSP isolates showed significantly higher susceptibility rates than PRSP isolates for all antibiotics (*P* < 0.0001) except the fluoroquinolones that showed excellent activity irrespective of penicillin susceptibility. PSSP isolates also had significantly higher susceptibility than PISP isolates to cefaclor, cefdinir, cefpodoxime, macrolides, tetracyclines and trimethoprim/sulfamethoxazole. Susceptibility of PISP isolates to the remaining antibiotics was 100% for amoxicillin, amoxicillin/clavulanic acid, cefotaxime, ceftriaxone and 97.0%–98.5% for fluoroquinolones. Lower susceptibility rates were observed against PRSP isolates for all antibiotics (0%–67.6% susceptible), except levofloxacin (97.3% susceptible) and moxifloxacin (100% susceptible).

### Comparative susceptibility of *S. pneumoniae* collected in 2012–14 and 2018–21

**Figure 4.**
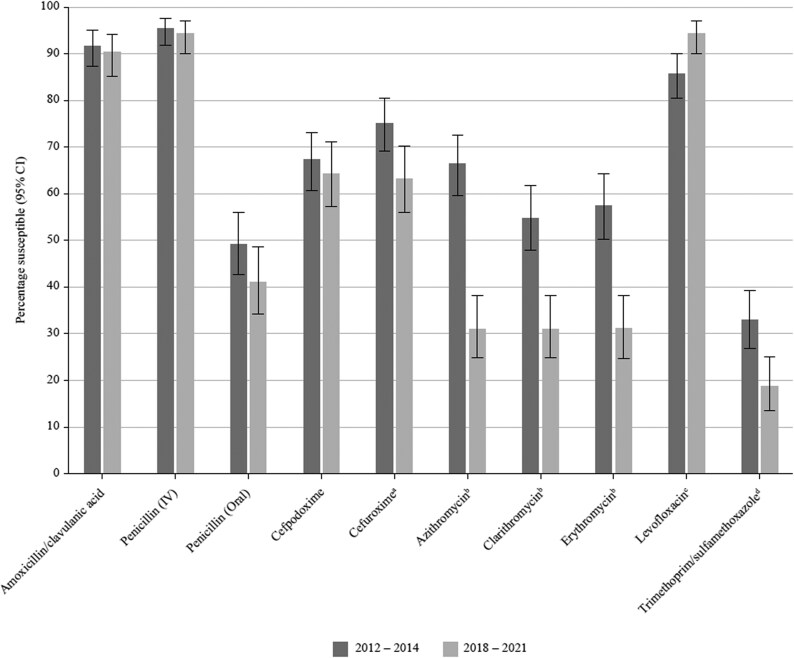
Comparison of antibiotic susceptibility rates of *S. pneumoniae* isolates from India collected in 2012–14 with isolates collected in 2018–21 (CLSI breakpoints). ^a^Susceptibility was significantly higher in 2012–14 than 2018–21 (*P* = 0.01). ^b^Susceptibility was significantly higher in 2012–14 than 2018–21 (*P* < 0.0001). ^c^Susceptibility was significantly lower in 2012–14 than 2018–21 (*P* = 0.01). ^d^Susceptibility was significantly higher in 2012–14 than 2018–21 (*P* = 0.002).

Data for the period 2012–14 have previously been published from the SOAR surveillance, and these data were compared for mutually tested antibiotics with the current study (2018–21) using CLSI breakpoints (Figure 4). There was no significant change in susceptibility to amoxicillin, amoxicillin/clavulanic acid, penicillin and cefpodoxime. However, a significant reduction in susceptibility to cefuroxime (*P* = 0.01), macrolides (*P* < 0.0001) and trimethoprim/sulfamethoxazole (*P* = 0.002) did occur between the two study periods. Interestingly, susceptibility to levofloxacin significantly increased further over this time period (*P* = 0.01) despite levofloxacin being 85.8% susceptible in 2012–14.

### *H. influenzae* isolates

A total of 171 *H. influenzae* isolates were collected from India. Most isolates originated from sputum (*n* = 158; 92.4%). The remaining isolates were from bronchoalveolar lavage (*n* = 8; 4.7%), endotracheal aspirate (*n* = 2; 1.2%), blood (*n* = 1; 0.6%) and unidentified specimens (*n* = 2; 1.2%). The majority of isolates (*n* = 123; 71.9%) came from adolescents and adults (aged 13–64 years), while isolates from elderly patients (aged ≥65 years) represented 26.9% (*n* = 46) and the remaining 1.2% (*n* = 2) were from paediatric patients (aged ≤12 years).

**Figure 5.**
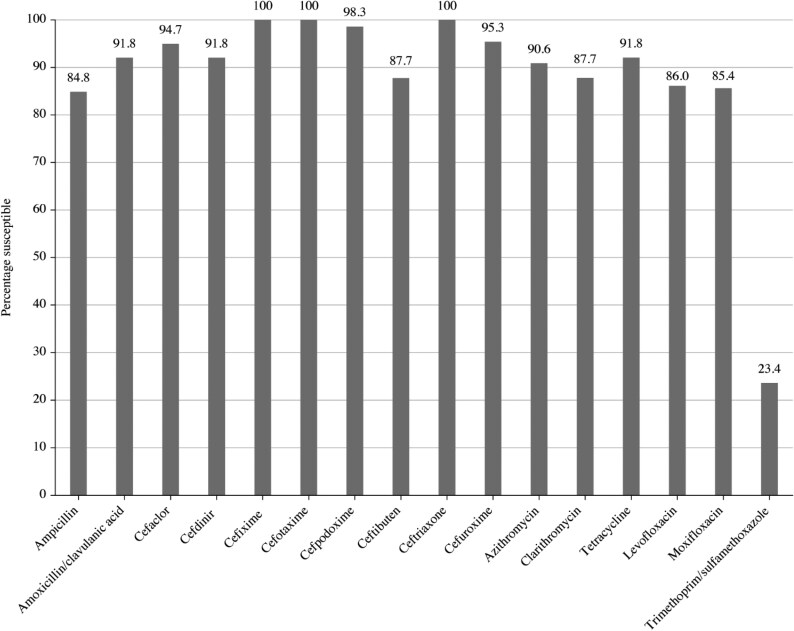
Antibiotic susceptibility rates of *H. influenzae* isolates (*n* = 171) from India based on CLSI breakpoints.

**Figure 6.**
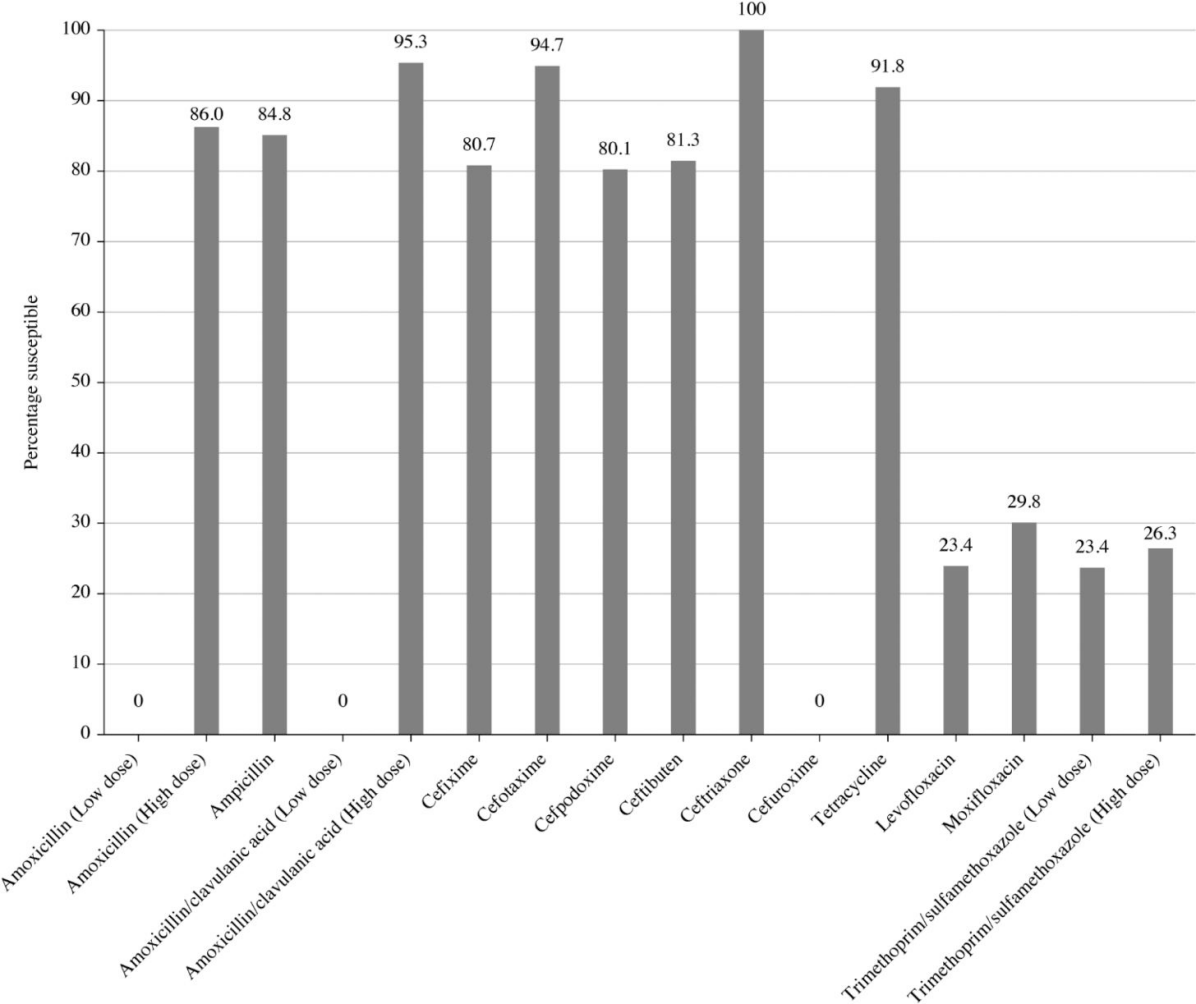
Antibiotic susceptibility rates of *H. influenzae* isolates (*n* = 171) from India based on EUCAST (dose-specific) breakpoints.

Summary MIC, susceptibility and MIC distribution data for all 171 *H. influenzae* isolates are shown in Tables 7–9 and S2 and Figures 5 and 6.

**Table 7.** MIC and susceptibility data for *H. influenzae* isolates (*n* = 171) from India using CLSI breakpoints

	MIC (mg/L)			CLSI susceptibility		
Antimicrobial	Range	50%	90%	S (%)	I (%)	R (%)
Amoxicillin	≤0.03 to 64	0.5	4	—	—	—
Ampicillin	≤0.03 to 128	0.25	2	84.8	5.8	9.4
Amoxicillin/clavulanic acid (2:1)	≤0.03 to 16	0.5	2	91.8	4.7	3.5
Cefaclor	≤0.25 to >32	2	8	94.7	1.2	4.1
Cefdinir	≤0.06 to >4	0.25	1	91.8	—	—
Cefixime	≤0.008 to 1	0.03	0.5	100	—	—
Cefotaxime	≤0.002 to 0.5	0.015	0.12	100	—	—
Cefpodoxime	≤0.015 to 4	0.06	2	98.3	—	—
Ceftibuten	≤0.008 to 4	0.06	4	87.7	—	—
Ceftriaxone	≤0.001 to 0.12	0.004	0.06	100	—	—
Cefuroxime	≤0.03 to >16	0.5	2	95.3	1.2	3.5
Azithromycin	≤0.12 to >8	1	4	90.6	—	—
Clarithromycin	≤0.25 to >32	4	16	87.7	3.5	8.8
Tetracycline	≤0.12 to 16	0.25	2	91.8	6.4	1.8
Levofloxacin	0.008 to >8	0.5	>8	86.0	—	—
Moxifloxacin	≤0.004 to >8	0.5	8	85.4	—	—
Trimethoprim/sulfamethoxazole	≤0.008 to >8	8	>8	23.4	5.3	71.3

—, not applicable; I, intermediate; R, resistant; S, susceptible.

**Table 8.** MIC and susceptibility data for *H. influenzae* isolates (*n* = 171) from India using EUCAST (dose-specific) breakpoints

	MIC (mg/L)			EUCAST susceptibility		
Antimicrobial	Range	50%	90%	S (%)	I (%)	R (%)
Amoxicillin (0.5 g × 3 oral)	≤0.03 to 64	0.5	4	0	86.0	14.0
Amoxicillin (0.75–1 g × 3 oral)	≤0.03 to 64	0.5	4	86.0	—	14.0
Ampicillin	≤0.03 to 128	0.25	2	84.8	—	15.2
Amoxicillin/clavulanic acid (0.5 g/0.125 g × 3 oral)	≤0.03 to 8	0.5	2	0	95.3	4.7
Amoxicillin/clavulanic acid (0.875 g/0.125 g × 3 oral)	≤0.03 to 8	0.5	2	95.3	—	4.7
Cefaclor	≤0.25 to >32	2	8	—	—	—
Cefdinir	≤0.06 to >4	0.25	1	—	—	—
Cefixime	≤0.008 to 1	0.03	0.5	80.7	—	19.3
Cefotaxime	≤0.002 to 0.5	0.02	0.12	94.7	—	5.3
Cefpodoxime	≤0.015 to 4	0.06	2	80.1	—	19.9
Ceftibuten	≤0.008 to 4	0.06	4	81.3	—	18.7
Ceftriaxone	≤0.001 to 0.12	0	0.06	100	—	0
Cefuroxime	≤0.03 to >16	0.5	2	0	74.3	25.7
Azithromycin	≤0.12 to >8	1	4	—	—	—
Clarithromycin	≤0.25 to >32	4	16	—	—	—
Tetracycline	≤0.12 to 16	0.25	2	91.8	—	8.2
Levofloxacin	0.008 to >8	0.5	>8	23.4	—	76.6
Moxifloxacin	≤0.004 to >8	0.5	8	29.8	—	70.2
Trimethoprim/sulfamethoxazole (0.16 g/0.8 g × 2 oral or IV)	≤0.008 to >8	8	>8	23.4	2.9	73.7
Trimethoprim/sulfamethoxazole (0.24 g/1.2 g × 2 oral or IV)	≤0.008 to >8	8	>8	26.3	—	73.7

—, not applicable; I, susceptible, increased exposure; R, resistant; S, susceptible.

**Table 9.** Summary MIC and susceptibility data for *H. influenzae* (*n* = 171) from India using PK/PD breakpoints

	MIC (mg/L)			PK/PD susceptibility
Antimicrobial	Range	50%	90%	S (%)
Amoxicillin (1.5 g/day)	≤0.03 to 64	0.5	4	86.0
Amoxicillin (4 g/day)	≤0.03 to 64	0.5	4	91.8
Amoxicillin/clavulanic acid (1.75 g/0.25 g/day adults; 45 mg/6.4 mg/kg/day children)	≤0.03 to 128	0.25	2	91.8
Amoxicillin/clavulanic acid (4 g/0.25 g/day adults; 90 mg/6.4 mg/kg/day children)	≤0.03 to 128	0.25	2	96.5
Ampicillin	≤0.03 to 16	0.5	2	—
Cefaclor	≤0.25 to >32	2	8	12.9
Cefdinir	≤0.06 to >4	0.25	1	55.0
Cefixime	≤0.008 to 1	0.03	0.5	100
Cefotaxime	≤0.002 to 0.5	0.015	0.12	—
Cefpodoxime	≤0.015 to 4	0.06	2	84.2
Ceftibuten	≤0.008 to 4	0.06	4	—
Ceftriaxone	≤0.001 to 0.12	0.004	0.06	100
Cefuroxime	≤0.03 to >16	0.5	2	74.3
Azithromycin	≤0.12 to >8	1	4	5.3
Clarithromycin	≤0.25 to >32	4	16	0.6
Tetracycline	≤0.12 to 16	0.25	2	—
Levofloxacin	0.008 to >8	0.5	>8	86.0
Moxifloxacin	≤0.004 to >8	0.5	8	85.4
Trimethoprim/sulfamethoxazole	≤0.008 to >8	8	>8	23.4

—, not applicable; PK/PD, pharmacokinetic/pharmacodynamic; S, susceptible.

### *H. influenzae* susceptibility

Most isolates of *H. influenzae* were β-lactamase negative (157/171, 91.8%). Within this population, 13 isolates were β-lactamase-negative ampicillin-resistant (BLNAR) by EUCAST breakpoints (ampicillin MIC ≥2 mg/L) and 5 by CLSI breakpoints (ampicillin MIC ≥4 mg/L). One β-lactamase-positive isolate was found to be ampicillin susceptible. Isolates from India were ≥84.8% susceptible to all antibiotics according to CLSI breakpoints, except for trimethoprim/sulfamethoxazole (23.4% susceptible). Similar results were obtained when using EUCAST breakpoints, provided high-dose regimens were used for amoxicillin and amoxicillin/clavulanate. However, cefuroxime susceptibility was 0%, 74.3% and 95.3% using EUCAST, PK/PD and CLSI breakpoints, respectively. Fluoroquinolone susceptibility was 23.4% (levofloxacin) and 29.8% (moxifloxacin) using EUCAST breakpoints. Macrolide breakpoints were not provided by EUCAST against *H. influenzae* but PK/PD breakpoints showed susceptibility of 5.3% (azithromycin) and 0.6% (clarithromycin). Otherwise, susceptibility was >80.0% using PK/PD breakpoints except for cefaclor (12.9% susceptible), cefdinir (55.0% susceptible) and trimethoprim/sulfamethoxazole (23.4% susceptible; Tables 7–9 and Figures 5 and 6).

### Comparative susceptibility of *H. influenzae* collected in 2012–14 and 2018–21

**Figure 7.**
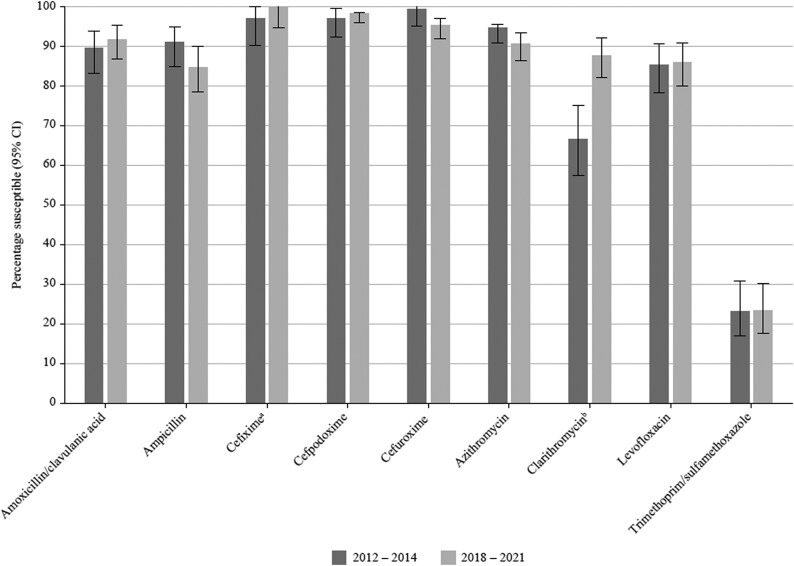
Comparison of antibiotic susceptibility rates of *H. influenzae* isolates from India collected in 2012–14 with isolates collected in 2018–21 (CLSI breakpoints). ^a^Susceptibility was significantly higher in 2018–21 than 2012–14 (*P* = 0.037). ^b^Susceptibility was significantly higher in 2018–21 than 2012–14 (*P* < 0.0001) but may be an artefact due to Etest breakpoints used in 2012–14.

There was no significant change in susceptibility to all antibiotics, except cefixime and clarithromycin when comparing data from 2012 to 2014 with data from 2018 to 2021 using CLSI breakpoints (Figure 7). Cefixime susceptibility was 97.0% in 2012–14 and increased to 100% in 2018–21 with statistical significance (*P* = 0.037). Clarithromycin activity also significantly increased between 2012–14 (66.7% susceptible) and 2018–21 (87.7% susceptible, *P* = 0.0001). However, it is important to note that MICs in the 2012–14 study were determined using Etest gradient strips with incubation in 5% CO_2_. It is known that high CO_2_ levels decrease macrolide activity due to a reduction in pH.^21^ For this reason, bioMérieux published adjusted Etest breakpoints for macrolides in CO_2_, which were used in the 2012–14 study.^15^

## Discussion

SOAR is an ongoing global surveillance study focusing on the two main CA-RTI pathogens, *S. pneumoniae* and *H. influenzae*, that has monitored numerous countries since 2002, including India. The data presented here are an analysis of the antibiotic susceptibility of *S. pneumoniae* and *H. influenzae* isolates collected from four hospital laboratories and two private laboratories in India between 2018 and 2021. A direct statistical comparison between the two study periods for common antimicrobial agents is also presented.

The penicillin susceptibility results for *S. pneumoniae* in India during 2018–21 clearly show that oral penicillin or low-dose IV penicillin is not an appropriate therapy for CA-RTIs because only 41.2% of pneumococci were susceptible using EUCAST low-dose IV or CLSI oral breakpoints. This low level of susceptibility to oral penicillin and low-dose IV penicillin was also observed in the previous (2012–14) SOAR analysis in India.^15,20^ Data from both CLSI and EUCAST guidelines, conversely, suggest that higher-dose IV penicillin could be a better option with susceptibility at 94.4%. CLSI and PK/PD breakpoints indicate a comparable level of susceptibility to amoxicillin, amoxicillin/clavulanic acid and third-generation cephalosporins (excluding cefdinir). However, susceptibility to β-lactams using EUCAST breakpoints was generally lower than when following CLSI guidelines, with susceptibility above 90% only observed with high-dose penicillin and high-dose ceftriaxone. These contrasting results are most likely based on different criteria used to determine breakpoints, but this has important practical implications for clinical laboratories and prescribing decisions. Susceptibility following all three guidelines indicated poor activity for macrolides, tetracyclines and trimethoprim/sulfamethoxazole but excellent activity for fluoroquinolones against *S. pneumoniae* from India. The MIC data from this study also highlighted a link between general antimicrobial susceptibility and susceptibility to penicillin, where PRSP isolates (CLSI oral breakpoints) were only susceptible to fluoroquinolones.

Outside of the SOAR study, antimicrobial susceptibility data for CA-RTI isolates from India are limited, but data from pneumococci isolated from a hospital in northern India, published in 2022, confirmed 11.8% susceptibility to trimethoprim/sulfamethoxazole and 92.6% susceptibility to penicillin using the CLSI IV dose breakpoint.^22^ However, levofloxacin susceptibility was lower (79.4%) and erythromycin susceptibility was higher (57.4%) than in the current study.^22^ The differences in susceptibility rates between the 2022 and the present study might be due to the different methods used (i.e. disc diffusion and broth microdilution, respectively).

The susceptibility of pneumococci using CLSI breakpoints for isolates previously collected in 2012–14 from India was compared with susceptibility from the current study (2018–21). As noted above for penicillin, there was no significant difference in susceptibility (CLSI breakpoints) between the two study periods for amoxicillin/clavulanic acid (91.8% versus 91.4%) or cefpodoxime, where susceptibility around 60% was maintained. Cefuroxime susceptibility significantly dropped from 2012–14 to 2018–21 to a level similar to that seen with cefpodoxime. Susceptibility to trimethoprim/sulfamethoxazole in 2012–14 (32.9%) decreased sharply to 18.6% in 2018–21, but the most significant reduction in activity was observed for the macrolides (i.e. from 66.3% to 33.1% for azithromycin). Interestingly, susceptibility to levofloxacin significantly increased over the same time period (85.8%–94.4%). The changes in pneumococcal susceptibility between the 2012–14 and the 2018–21 studies could reflect a shift in serotype prevalence due to the national introduction of the pneumococcal conjugate vaccine in 2017.^23^

Most *H. influenzae* from India were β-lactamase negative (91.8%) with 13 being BLNAR according to EUCAST breakpoints and 5 by CLSI breakpoints. Susceptibility to antibiotics was ≥84.8% by CLSI breakpoints, except for trimethoprim/sulfamethoxazole (23.4% susceptible). In most cases, susceptibility with EUCAST breakpoints was slightly lower than with CLSI or PK/PD breakpoints, but greater differences were observed with cefuroxime (0%, 95.3% and 74.3%, respectively), levofloxacin (23.4%, 86.0% and 86.0%, respectively) and moxifloxacin (29.8%, 85.4% and 85.4%, respectively). Macrolide susceptibility by PK/PD breakpoints was much lower than by CLSI breakpoints, the latter being in keeping with EUCAST where no breakpoints are given. Data from the large ATLAS surveillance interactive database for *H. influenzae* collected from 2018 to 2021 show that levofloxacin susceptibility by EUCAST breakpoints was 95.2% overall but only 25% in India.^24^ SOAR surveillance from 2012 to 2014 used older EUCAST levofloxacin breakpoints from 2015 (v5.0) where the susceptible breakpoint was ≤1 mg/L.^25^ However, a recalculation based on the MIC distributions from 2012 to 2014 using current breakpoints indicates levofloxacin susceptibility at 24%.^19^ The levofloxacin-resistant isolates by EUCAST breakpoints in India and Pakistan have non-WT MICs, but most would still be considered susceptible by CLSI. It is possible, therefore, that dominant levofloxacin-non-WT (but levofloxacin susceptible by CLSI breakpoints) isolates have been circulating in Asia since 2012. SOAR surveillance from 2012 to 2014 also indicated generally high antibiotic susceptibility with *H. influenzae*, except for trimethoprim/sulfamethoxazole.^15,20^ There was a statistically significant increase in susceptibility to cefixime (97.0%–100%) and clarithromycin (66.7%–87.7%) over this time period. However, the difference in clarithromycin susceptibility between the two periods may be an artefact due to non-standard MICs using Etests in CO_2_ and adjusted bioMérieux breakpoints used in the 2012–14 study.^15^ The results for azithromycin, however, did not show this effect despite being tested under the same conditions.

*S. pneumoniae* causes serious invasive disease including pneumonia, life-threatening septicaemia and meningitis; institution of the correct antibiotic on suspicion is of paramount importance for a favourable outcome. *H. influenzae* is also a causative agent of pneumonia and meningitis. In summary, based on the present surveillance data, for *S. pneumoniae* CA-RTIs, oral or low-dose IV penicillin therapy is inadequate, with only 41.2% of isolates susceptible, while higher-dose IV penicillin showed increased susceptibility of 94.4%. Fluoroquinolones and ceftriaxone demonstrated excellent activity (89.8%–96.6% susceptibility). Over time, however, there was a significant reduction in pneumococcal susceptibility to macrolides. In *H. influenzae*, despite some methodological differences between CLSI, EUCAST and PK/PD, susceptibility generally remained high. Notable changes over time in susceptibility to cefpodoxime or cefuroxime (reduced) and to cefixime or clarithromycin (increased) were seen. Ceftriaxone and cefotaxime were most active according to CLSI and EUCAST breakpoints. According to PK/PD, susceptibility to ceftriaxone was also 100% but no breakpoints are available for cefotaxime. Most isolates (91.8%) were β-lactamase negative.

In addition, the differences in susceptibility between blood and non-blood isolates were globally assessed; the data are not shown in this manuscript, but they were presented at ESCMID Global 2025.^26^  *S. pneumoniae* and *H. influenzae* susceptibility rates for most antibiotics were comparable between blood and non-blood isolates. However, some non-blood isolates were less susceptible than blood isolates, specifically, penicillin (oral), trimethoprim/sulfamethoxazole and second-generation cephalosporins (*S. pneumoniae*) and aminopenicillins, trimethoprim/sulfamethoxazole and levofloxacin (*H. influenzae*).

A limitation of the past and present SOAR studies is a possible bias in the susceptibility rates due to eventual antibiotic administration prior to sample collection. An antibiotic pretreatment could have selected for more resistant bacterial populations. However, as a real-world assessment of antimicrobial resistance, the SOAR program monitors the actual resistance landscape faced by clinicians globally. Therefore, patients with antibiotic pretreatment were not excluded, as in many regions empirical antibiotic therapy is initiated prior to sample collection. Only ceftriaxone had sufficient activity to cover both CA-RTI pathogens using the three breakpoint standards. It is recommended to use standardized methods for the isolation of fastidious organisms such as *H. influenzae* and *S. pneumoniae* and appropriate antimicrobial susceptibility testing as a first step to generate a representative database for antibiotics in community settings, considering different risk groups and patient categories. This will help establish a baseline to monitor further trends in antibiotic resistance for both pathogens. This study emphasizes the importance of continued surveillance of antibiotic susceptibility in India to regularly assess any future changes and the impact of differing susceptibility criteria on clinical decisions.

## Supplementary Material

dkaf285_Supplementary_Data

## Supplementary data

Tables S1 and S2 are available as Supplementary data at *JAC* Online.
