# Supplementary material for: Results from the Survey of Antibiotic Resistance (SOAR) 2018–21 in India: data based on CLSI, EUCAST (dose-specific) and pharmacokinetic/pharmacodynamic (PK/PD) breakpoints
Source: J Antimicrob Chemother. 2025 Nov 24;80(Suppl 3):iii38–52. doi: 10.1093/jac/dkaf285 (PMC12641134; doi:10.1093/jac/dkaf285)
Supplement: dkaf285_Supplementary_Data [file dkaf285_supplementary_data.docx]

**Results from the Survey of Antibiotic Resistance (SOAR) 2018 – 21 in India: data based on CLSI, EUCAST (dose-specific) and pharmacokinetic/pharmacodynamic (PK/PD) breakpoints**

**Authors:** Didem TORUMKUNEY^1^, Balaji VEERARAGHAVAN^2^, Niranjan PATIL^3^, Mary DIAS^4^, Geeti MAHESHWARI^5^, Bhaskar Narayan CHAUDHURI^6^, Ujjwayini RAY^7^, Stephen HAWSER^8^, Subhashri KUNDU^9^, Anand MANOHARAN^10*^

**Affiliations:** ^1^GSK, London, UK; ^2^Department of Clinical Microbiology, Christian Medical College, Vellore, India; ^3^Metropolis Healthcare Private Limited, Vidyavihar West, Mumbai, India; ^4^Department of Microbiology and Division of Infectious Diseases, St John's Medical College Hospital, Bangalore, India; ^5^Department of Microbiology and Molecular Sciences, Toprani Advanced Lab Systems, Vadodara, India; ^6^Peerless Hospital, Kolkata, India; ^7^Apollo Multispecialty Hospitals, Kolkata, India; ^8^IHMA Europe Sàrl, Monthey, Switzerland; ^9^GSK, Singapore; ^10^Infectious Diseases Medical & Scientific Affairs, GSK, Mumbai, India

*Corresponding author. E-mail: [anand.x.manoharan@gsk.com](mailto:anand.x.manoharan@gsk.com)

**Running title:** Survey of Antibiotic Resistance (SOAR) in India in 2018 – 21

**Supplementary Table 1.** MIC distribution data for *S. pneumoniae* isolates (*n* = 177) from India

|  | | | | Number of isolates at MIC (mg/L) | | | | | | | | | | | | | | | | | | | | | |
| --- | --- | --- | --- | --- | --- | --- | --- | --- | --- | --- | --- | --- | --- | --- | --- | --- | --- | --- | --- | --- | --- | --- | --- | --- | --- |
| Antimicrobial |  | | ≤0.008 | | | ≤0.015 | 0.015 | ≤0.03 | 0.03 | 0.06 | 0.12 | ≤0.25 | 0.25 | 0.5 | 1 | 2 | | 4 | | >4 | | 8 | >8 | 16 | >16 |
| AMX | N | | – | | | – | 26 | – | 46 | 6 | 12 | – | 18 | 7 | 16 | 29 | | 7 | | – | | 10 | – | – | – |
|  | | Cum. % | – | | | – | 14.7 | – | 40.7 | 44.1 | 50.9 | – | 61.1 | 65.1 | 74.1 | 90.5 | | 94.5 | | – | | 100 | – | – | – |
|  | % | | – | | | – | 14.7 | – | 26 | 3.4 | 6.8 | – | 10.2 | 4 | 9 | 16.4 | | 4 | | – | | 5.6 | – | – | – |
| AMC (2:1) | N | | 11 | | | – | 13 | – | 49 | 4 | 12 | – | 21 | 4 | 18 | 28 | | 7 | | – | | 10 | – | – | – |
|  | Cum. % | | 6.2 | | | – | 13.5 | – | 41.2 | 43.5 | 50.3 | – | 62.2 | 64.5 | 74.7 | 90.5 | | 94.5 | | – | | 100 | – | – | – |
|  | % | | 6.2 | | | – | 7.3 | – | 27.7 | 2.3 | 6.8 | – | 11.9 | 2.3 | 10.2 | 15.8 | | 4 | | – | | 5.6 | – | – | – |
| AMC | N | | 19 | | | – | 1 | - | 24 | 29 | 10 | – | 13 | 10 | 12 | 9 | | 14 | | – | | 28 | 8 | – | – |
| [2 mg/L] | Cum. % | | 10.7 | | | – | 11.3 | - | 24.9 | 41.3 | 46.9 | – | 54.2 | 59.8 | 66.6 | 71.7 | | 79.6 | | – | | 95.4 | 100 | – | – |
|  | % | | 10.7 | | | – | 0.6 | - | 13.6 | 16.4 | 5.6 | – | 7.3 | 5.6 | 6.8 | 5.1 | | 7.9 | | – | | 15.8 | 4.5 | – | – |
| AZM | N | | – | | | 1 | – | – | 3 | 29 | 17 | – | 5 | – | – | 9 | | 17 | | – | | 13 | – | 12 | 71 |
|  | Cum. % | | – | | | 0.6 | – | – | 2.3 | 18.7 | 28.3 | – | 31.1 | – | – | 36.2 | | 45.8 | | – | | 53.1 | – | 59.9 | 100 |
|  | % | | – | | | 0.6 | – | – | 1.7 | 16.4 | 9.6 | – | 2.8 | – | – | 5.1 | | 9.6 | | – | | 7.3 | – | 6.8 | 40.1 |
| CEC | N | | – | | | – | – | – | – | – | – | – | 2 | 47 | 30 | 20 | | 3 | | 75 | | – | – | – | – |
|  | Cum. % | | – | | | – | – | – | – | – | – | – | 1.1 | 27.7 | 44.6 | 55.9 | | 57.6 | | 100 | | – | – | – | – |
|  | % | | – | | | – | – | – | – | – | – | – | 1.1 | 26.6 | 16.9 | 11.3 | | 1.7 | | 42.4 | | – | – | – | – |
| CDR | N | | – | | | – | – | – | 8 | 40 | 18 | – | 20 | 18 | 10 | 11 | | 23 | | – | | 28 | 1 | – | – |
|  | Cum. % | | – | | | – | – | – | 4.5 | 27.1 | 37.3 | – | 48.6 | 58.8 | 64.4 | 70.6 | | 83.6 | | – | | 99.4 | 100 | – | – |
|  | % | | – | | | – | – | – | 4.5 | 22.6 | 10.2 | – | 11.3 | 10.2 | 5.6 | 6.2 | | 13.0 | | – | | 15.8 | 0.6 | – | – |
| CFM | N | | – | | | – | – | – | – | – | – | 43 | – | 12 | 13 | 23 | | 23 | | – | | 9 | – | 34 | 20 |
|  | Cum. % | | – | | | – | – | – | – | – | – | 24.3 | – | 31.1 | 38.4 | 51.4 | | 64.4 | | – | | 69.5 | – | 88.7 | 100 |
|  | % | | – | | | – | – | – | – | – | – | 24.3 | – | 6.8 | 7.3 | 13 | | 13 | | – | | 5.1 | – | 19.2 | 11.3 |
| CTX | N | | 1 | | | – | 33 | – | 16 | 8 | 27 | – | 23 | 17 | 40 | 12 | | – | | – | | – | – | – | – |
|  | Cum. % | | 0.6 | | | – | 19.2 | – | 28.2 | 32.7 | 48.0 | – | 61.0 | 70.6 | 93.2 | 100 | | – | | – | | – | – | – | – |
|  | % | | 0.6 | | | – | 18.6 | – | 9.0 | 4.5 | 15.3 | – | 13.0 | 9.6 | 22.6 | 6.8 | | – | | – | | – | – | – | – |
| CPD | N | | – | | | 5 | – | – | 41 | 10 | 14 | – | 28 | 16 | 10 | 30 | | 21 | | 2 | | – | – | – | – |
|  | Cum. % | | – | | | 2.8 | – | – | 26 | 31.6 | 39.5 | – | 55.3 | 64.3 | 69.9 | 86.8 | | 98.7 | | 100 | | – | – | – | – |
|  | % | | – | | | 2.8 | – | – | 23.2 | 5.6 | 7.9 | – | 15.8 | 9.0 | 5.6 | 16.9 | | 11.9 | | 1.1 | | – | – | – | – |
| CTB | N | | – | | | – | – | – | – | – | – | – | – | – | – | 3 | | 39 | | – | | 8 | – | 15 | 112 |
|  | Cum. % | | – | | | – | – | – | – | – | – | – | – | – | – | 1.7 | | 23.7 | | – | | 28.2 | – | 36.7 | 100 |
|  | % | | – | | | – | – | – | – | – | – | – | – | – | – | 1.7 | | 22.0 | | – | | 4.5 | – | 8.5 | 63.3 |
| CRO | N | | – | | | – | 16 | – | 31 | 8 | 18 | - | 27 | 19 | 40 | 18 | | – | | – | | – | – | – | – |
|  | Cum. % | | – | | | – | 9 | – | 26.5 | 31.0 | 41.2 | - | 56.5 | 67.2 | 89.8 | 100 | | – | | – | | – | – | – | – |
|  | % | | – | | | – | 9.0 | – | 17.5 | 4.5 | 10.2 | - | 15.3 | 10.7 | 22.6 | 10.2 | | – | | – | | – | – | – | – |
| CXM | N | | 1 | | | – | 13 | – | 26 | 9 | 13 | – | 21 | 29 | – | 7 | | 33 | | – | | 25 | – | – | – |
|  | Cum. % | | 0.6 | | | – | 7.9 | – | 22.6 | 27.7 | 35 | – | 46.9 | 63.3 | – | 67.3 | | 85.9 | | – | | 100 | – | – | – |
|  | % | | 0.6 | | | – | 7.3 | – | 14.7 | 5.1 | 7.3 | – | 11.9 | 16.4 | – | 4 | | 18.6 | | – | | 14.1 | – | – | – |
| CLR | N | | – | | | 13 | – | – | 37 | 5 | – | – | – | 5 | 11 | 19 | | 9 | | – | | 11 | – | 3 | 64 |
|  | Cum. % | | – | | | 7.3 | – | – | 28.2 | 31.0 | – | – | – | 33.8 | 40.0 | 50.7 | | 55.8 | | – | | 62.0 | – | 63.7 | 100 |
|  | % | | – | | | 7.3 | – | – | 20.9 | 2.8 | – | – | – | 2.8 | 6.2 | 10.7 | | 5.1 | | – | | 6.2 | – | 1.7 | 36.2 |
| DOX | N | | – | | | – | – | – | 1 | 30 | 18 | – | 1 | 1 | 3 | 10 | | 47 | | 66 | | – | – | – | – |
|  | Cum. % | | – | | | – | – | – | 0.6 | 17.5 | 27.7 | – | 28.3 | 28.9 | 30.6 | 36.2 | | 62.8 | | 100 | | – | – | – | – |
|  | % | | – | | | – | – | – | 0.6 | 16.9 | 10.2 | – | 0.6 | 0.6 | 1.7 | 5.6 | | 26.6 | | 37.3 | | – | – | – | – |
| ERY | N | | – | | | 3 | – | – | 28 | 22 | 2 | – | – | – | 2 | 20 | | 14 | | – | | 9 | – | 10 | 67 |
|  | Cum. % | | – | | | 1.7 | – | – | 17.5 | 29.9 | 31.0 | – | – | – | 32.1 | 43.4 | | 51.3 | | – | | 56.4 | – | 62.0 | 100 |
|  | % | | – | | | 1.7 | – | – | 15.8 | 12.4 | 1.1 | – | – | – | 1.1 | 11.3 | | 7.9 | | – | | 5.1 | – | 5.6 | 37.9 |
| LVX | N | | – | | | – | – | – | – | – | – | – | 1 | 13 | 148 | 5 | | 1 | | – | | 3 | 6 | – | – |
|  | Cum. % | | – | | | – | – | – | – | – | – | – | 0.6 | 7.9 | 91.5 | 94.3 | | 94.9 | | – | | 96.6 | 100 | – | – |
|  | % | | – | | | – | – | – | – | – | – | – | 0.6 | 7.3 | 83.6 | 2.8 | | 0.6 | | – | | 1.7 | 3.4 | – | – |
| MXF | N | | – | | | – | – | 3 | – | 29 | 136 | – | 2 | 1 | – | 6 | | – | | – | | – | – | – | – |
|  | Cum. % | | – | | | – | – | 1.7 | – | 18.1 | 94.9 | – | 96.0 | 96.6 | – | 100 | | – | | – | | – | – | – | – |
|  | % | | – | | | – | – | 1.7 | – | 16.4 | 76.8 | – | 1.1 | 0.6 | – | 3.4 | | – | | – | | – | – | – | – |
| PEN | N | | 1 | | | – | 29 | – | 28 | 15 | 11 | – | 23 | 8 | 25 | 27 | | 10 | | – | | – | – | – | – |
|  | Cum. % | | 0.6 | | | – | 17.0 | – | 32.8 | 41.3 | 47.5 | – | 60.5 | 65.0 | 79.1 | 94.4 | | 100 | | – | | – | – | – | – |
|  | % | | 0.6 | | | – | 16.4 | – | 15.8 | 8.5 | 6.2 | – | 13 | 4.5 | 14.1 | 15.3 | | 5.6 | | – | | – | – | – | – |
| TET | N | | – | | | – | – | – | – | – | 30 | – | 19 | – | 1 | 1 | | 4 | | 122 | | – | – | – | – |
|  | Cum. % | | – | | | – | – | – | – | – | 16.9 | – | 27.6 | – | 28.2 | 28.8 | | 31.1 | | 100 | | – | – | – | – |
|  | % | | – | | | – | – | – | – | – | 16.9 | – | 10.7 | – | 0.6 | 0.6 | | 2.3 | | 68.9 | | – | – | – | – |
| SXT | N | | – | | | – | – | – | – | – | 3 | – | 16 | 14 | 16 | 7 | | 74 | | – | | 39 | 8 | – | – |
|  | Cum. % | | – | | | – | – | – | – | – | 1.7 | – | 10.7 | 18.6 | 27.6 | 31.6 | | 73.4 | | – | | 95.4 | 100 | – | – |
|  | % | | – | | | – | – | – | – | – | 1.7 | – | 9.0 | 7.9 | 9.0 | 4.0 | | 41.8 | | – | | 22.0 | 4.5 | – | – |
|  | | | |  |  |  |  |  |  |  |  |  |  |  |  | |  | |  | |  |  |  |  |  |

–, not applicable; AMC, amoxicillin/clavulanic acid; AMX, amoxicillin; AZM, azithromycin; CDR, cefdinir; CEC, cefaclor; CFM, cefixime; CLR, clarithromycin; CPD, cefpodoxime; CRO, ceftriaxone; CTB, ceftibuten; CTX, cefotaxime; Cum., cumulative; CXM, cefuroxime; DOX, doxycycline; ERY, erythromycin; LVX, levofloxacin; MXF, moxifloxacin; PEN, penicillin; SXT, trimethoprim/sulfamethoxazole; TET, tetracycline.

Bold vertical bars in table correspond to the CLSI-susceptible breakpoints.

**Supplementary Table 2.** MIC distribution data for *H. influenzae* isolates (*n* = 171) from India

|  |  | Number of isolates at MIC (mg/L) | | | | | | | | | | | | | | | | | | | | | | | | | | | | | |
| --- | --- | --- | --- | --- | --- | --- | --- | --- | --- | --- | --- | --- | --- | --- | --- | --- | --- | --- | --- | --- | --- | --- | --- | --- | --- | --- | --- | --- | --- | --- | --- |
| Antimicrobial |  | ≤0.001 | ≤0.002 | 0.002 | ≤0.004 | 0.004 | ≤0.008 | 0.008 | ≤0.015 | 0.015 | ≤0.03 | 0.03 | ≤0.06 | 0.06 | ≤0.12 | 0.12 | ≤0.25 | 0.25 | 0.5 | 1 | 2 | 4 | >4 | 8 | >8 | 16 | >16 | 32 | >32 | 64 | 128 |
| AMX | N | – | – | – | – | – | – | – | – | – | 7 | – | – | – | – | 3 | – | 35 | 52 | 16 | 34 | 10 | – | 9 | – | 3 | – | – | – | 2 | – |
|  | Cum. % | – | – | – | – | – | – | – | – | – | 4.1 | – | – | – | – | 5.9 | – | 26.4 | 56.8 | 66.2 | 86.1 | 91.9 | – | 97.2 | – | 99 | – | – | – | 100 | – |
|  | % | – | – | – | – | – | – | – | – | – | 4.1 | – | – | – | – | 1.8 | – | 20.5 | 30.4 | 9.4 | 19.9 | 5.8 | – | 5.3 | – | 1.8 | – | – | – | 1.2 | – |
| AMC (2:1) | N | – | – | – | – | – | – | – | – | – | 4 | – | – | 2 | – | 3 | – | 14 | 72 | 29 | 33 | 8 | – | 5 | – | 1 | – | – | – | – | – |
|  | Cum. % | – | – | – | – | – | – | – | – | – | 2.3 | – | – | 3.5 | – | 5.3 | – | 13.5 | 55.6 | 72.6 | 91.9 | 96.6 | – | 99.5 | – | 100 | – | – | – | – | – |
|  | % | – | – | – | – | – | – | – | – | – | 2.3 | – | – | 1.2 | – | 1.8 | – | 8.2 | 42.1 | 17.0 | 19.3 | 4.7 | – | 2.9 | – | 0.6 | – | – | – | – | – |
| AMC | N | – | – | – | – | – | – | – | – | – | 8 | – | – | – | – | 11 | – | 49 | 47 | 16 | 32 | 4 | – | 4 | – | – | – | – | – | – | – |
| [2 mg/L] | Cum. % | – | – | – | – | – | – | – | – | – | 4.7 | – | – | – | – | 11.1 | – | 39.8 | 67.3 | 76.7 | 95.4 | 97.7 | – | 100 | – | – | – | – | – | – | – |
|  | % | – | – | – | – | – | – | – | – | – | 4.7 | – | – | – | – | 6.4 | – | 28.7 | 27.5 | 9.4 | 18.7 | 2.3 | – | 2.3 | – | – | – | – | – | – | – |
| AMP | N | – | – | – | – | – | – | – | – | – | 8 | – | – | 1 | – | 35 | – | 45 | 14 | 42 | 10 | 8 | – | 5 | – | 1 | – | – | – | 1 | 1 |
|  | Cum. % | – | – | – | – | – | – | – | – | – | 4.7 | – | – | 5.3 | – | 25.8 | – | 52.1 | 60.3 | 84.9 | 90.7 | 95.4 | – | 98.3 | – | 98.9 | – | – | – | 99.5 | 100 |
|  | % | – | – | – | – | – | – | – | – | – | 4.7 | – | – | 0.6 | – | 20.5 | – | 26.3 | 8.2 | 24.6 | 5.8 | 4.7 | – | 2.9 | – | 0.6 | – | – | – | 0.6 | 0.6 |
| AZM | N | – | – | – | – | – | – | – | – | – | – | – | – | – | 9 | – | – | 14 | 51 | 63 | 14 | 4 | – | 3 | 13 | – | – | – | – | – | – |
|  | Cum. % | – | – | – | – | – | – | – | – | – | – | – | – | – | 5.3 | – | – | 13.5 | 43.3 | 80.1 | 88.3 | 90.6 | – | 92.4 | 100 | – | – | – | – | – | – |
|  | % | – | – | – | – | – | – | – | – | – | – | – | – | – | 5.3 | – | – | 8.2 | 29.8 | 36.8 | 8.2 | 2.3 | – | 1.8 | 7.6 | – | – | – | – | – | – |
| CEC | N | – | – | – | – | – | – | – | – | – | – | – | – | – | – | – | 3 | – | 19 | 39 | 29 | 53 | – | 19 | – | 2 | – | 2 | 5 | – | – |
|  | Cum. % | – | – | – | – | – | – | – | – | – | – | – | – | – | – | – | 1.8 | – | 12.9 | 35.7 | 52.7 | 83.7 | – | 94.8 | – | 96 | – | 97.2 | 100 | – | – |
|  | % | – | – | – | – | – | – | – | – | – | – | – | – | – | – | – | 1.8 | – | 11.1 | 22.8 | 17.0 | 31.0 | – | 11.1 | – | 1.2 | – | 1.2 | 2.9 | – | – |
| CDR | N | – | – | – | – | – | – | – | – | – | – | – | 5 | – | – | 35 | – | 54 | 40 | 23 | 8 | 4 | 2 | – | – | – | – | – | – | – | – |
|  | Cum. % | – | – | – | – | – | – | – | – | – | – | – | 2.9 | – | – | 23.4 | – | 55.0 | 78.4 | 91.9 | 96.6 | 98.9 | 100 | – | – | – | – | – | – | – | – |
|  | % | – | – | – | – | – | – | – | – | – | – | – | 2.9 | – | – | 20.5 | – | 31.6 | 23.4 | 13.5 | 4.7 | 2.3 | 1.2 | – | – | – | – | – | – | – | – |
| CFM | N | – | – | – | – | – | 5 | – | – | 45 | – | 68 | – | 13 | – | 7 | – | 10 | 14 | 9 | – | – | – | – | – | – | – | – | – | – | – |
|  | Cum. % | – | – | – | – | – | 2.9 | – | – | 29.2 | – | 69.0 | – | 76.6 | – | 80.7 | – | 86.5 | 94.7 | 100 | – | – | – | – | – | – | – | – | – | – | – |
|  | % | – | – | – | – | – | 2.9 | – | – | 26.3 | – | 39.8 | – | 7.6 | – | 4.1 | – | 5.8 | 8.2 | 5.3 | – | – | – | – | – | – | – | – | – | – | – |
| CTX | N | – | 42 | – | – | 12 | – | 22 | – | 20 | – | 33 | – | 15 | – | 18 | – | 2 | 7 | – | – | – | – | – | – | – | – | – | – | – | – |
|  | Cum. % | – | 24.6 | – | – | 31.6 | – | 44.5 | – | 56.2 | – | 75.5 | – | 84.3 | – | 94.8 | – | 96 | 100 | – | – | – | – | – | – | – | – | – | – | – | – |
|  | % | – | 24.6 | – | – | 7.0 | – | 12.9 | – | 11.7 | – | 19.3 | – | 8.8 | – | 10.5 | – | 1.2 | 4.1 | – | – | – | – | – | – | – | – | – | – | – | – |
| CPD | N | – | – | – | – | – | – | – | 10 | – | – | 45 | – | 38 | – | 39 | – | 5 | 7 | 8 | 16 | 3 | – | – | – | – | – | – | – | – | – |
|  | Cum. % | – | – | – | – | – | – | – | 5.8 | – | – | 32.1 | – | 54.3 | – | 77.1 | – | 80 | 84.1 | 88.8 | 98.2 | 100 | – | – | – | – | – | – | – | – | – |
|  | % | – | – | – | – | – | – | – | 5.8 | – | – | 26.3 | – | 22.2 | – | 22.8 | – | 2.9 | 4.1 | 4.7 | 9.4 | 1.8 | – | – | – | – | – | – | – | – | – |
| CTB | N | – | – | – | – | – | 2 | – | – | 1 | – | 46 | – | 38 | – | 8 | – | 35 | 3 | 6 | 11 | 21 | – | – | – | – | – | – | – | – | – |
|  | Cum. % | – | – | – | – | – | 1.2 | – | – | 1.8 | – | 28.7 | – | 50.9 | – | 55.6 | – | 76.1 | 77.9 | 81.4 | 87.8 | 100 | – | – | – | – | – | – | – | – | – |
|  | % | – | – | – | – | – | 1.2 | – | – | 0.6 | – | 26.9 | – | 22.2 | – | 4.7 | – | 20.5 | 1.8 | 3.5 | 6.4 | 12.3 | – | – | – | – | – | – | – | – | – |
| CRO | N | 26 | – | 28 | – | 35 | – | 16 | – | 34 | – | 13 | – | 9 | – | 10 | – | – | – | – | – | – | – | – | – | – | – | – | – | – | – |
|  | Cum. % | 15.2 | – | 31.6 | – | 52.1 | – | 61.5 | – | 81.4 | – | 89.0 | – | 94.3 | – | 100 | – | – | – | – | – | – | – | – | – | – | – | – | – | – | – |
|  | % | 15.2 | – | 16.4 | – | 20.5 | – | 9.4 | – | 19.9 | – | 7.6 | – | 5.3 | – | 5.8 | – | – | – | – | – | – | – | – | – | – | – | – | – | – | – |
| CXM | N | – | – | – | – | – | – | – | – | – | 2 | – | – | 6 | – | 4 | – | 29 | 53 | 33 | 32 | 4 | – | 2 | – | 3 | 3 | – | – | – | – |
|  | Cum. % | – | – | – | – | – | – | – | – | – | 1.2 | – | – | 4.7 | – | 7 | – | 24 | 55 | 74.3 | 93 | 95.3 | – | 96.5 | – | 98.3 | 100 | – | – | – | – |
|  | % | – | – | – | – | – | – | – | – | – | 1.2 | – | – | 3.5 | – | 2.3 | – | 17.0 | 31.0 | 19.3 | 18.7 | 2.3 | – | 1.2 | – | 1.8 | 1.8 | – | – | – | – |
| CLR | N | – | – | – | – | – | – | – | – | – | – | – | – | – | – | – | 1 | – | 2 | 2 | 12 | 85 | – | 48 | – | 6 | – | 3 | 12 | – | – |
|  | Cum. % | – | – | – | – | – | – | – | – | – | – | – | – | – | – | – | 0.6 | – | 1.8 | 3.0 | 10.0 | 59.7 | – | 87.8 | – | 91.3 | – | 93.1 | 100 | – | – |
|  | % | – | – | – | – | – | – | – | – | – | – | – | – | – | – | – | 0.6 | – | 1.2 | 1.2 | 7.0 | 49.7 | – | 28.1 | – | 3.5 | – | 1.8 | 7.0 | – | – |
| LVX | N | – | – | – | – | – | – | 3 | – | 17 | – | 6 | – | 14 | – | 10 | – | 8 | 76 | 12 | 1 | 1 | – | 3 | 20 | – | – | – | – | – | – |
|  | Cum. % | – | – | – | – | – | – | 1.8 | – | 11.7 | – | 15.2 | – | 23.4 | – | 29.2 | – | 33.9 | 78.3 | 85.3 | 85.9 | 86.5 | – | 88.3 | 100 | – | – | – | – | – | – |
|  | % | – | – | – | – | – | – | 1.8 | – | 9.9 | – | 3.5 | – | 8.2 | – | 5.8 | – | 4.7 | 44.4 | 7.0 | 0.6 | 0.6 | – | 1.8 | 11.7 | – | – | – | – | – | – |
| MXF | N | – | – | – | 2 | – | – | 2 | – | 15 | – | 6 | – | 10 | – | 16 | – | 7 | 55 | 33 | 1 | 2 | – | 15 | 7 | – | – | – | – | – | – |
|  | Cum. % | – | – | – | 1.2 | – | – | 2.4 | – | 11.2 | – | 14.7 | – | 20.5 | – | 29.9 | – | 34.0 | 66.2 | 85.5 | 86.1 | 87.3 | – | 96.1 | 100 | – | – | – | – | – | – |
|  | % | – | – | – | 1.2 | – | – | 1.2 | – | 8.8 | – | 3.5 | – | 5.8 | – | 9.4 | – | 4.1 | 32.2 | 19.3 | 0.6 | 1.2 | – | 8.8 | 4.1 | – | – | – | – | – | – |
| TET | N | – | – | – | – | – | – | – | – | – | – | – | – | – | 8 | – | – | 134 | 8 | 2 | 5 | 11 | – | 2 | – | 1 | – | – | – | – | – |
|  | Cum. % | – | – | – | – | – | – | – | – | – | – | – | – | – | 4.7 | – | – | 82.8 | 87.5 | 88.7 | 92.2 | 98.6 | – | 99.8 | – | 100 | – | – | – | – | – |
|  | % | – | – | – | – | – | – | – | – | – | – | – | – | – | 4.7 | – | – | 78.1 | 4.7 | 1.2 | 3.5 | 6.4 | – | 1.2 | – | 0.6 | – | – | – | – | – |
| SXT | N | – | – | – | – | – | 1 | – | – | 1 | – | 2 | – | 8 | – | 17 | – | 10 | 1 | 5 | 4 | 12 | – | 74 | 36 | – | – | – | – | – | – |
|  | Cum. % | – | – | – | – | – | 0.6 | – | – | 1.2 | – | 2.4 | – | 7.1 | – | 17 | – | 22.8 | 23.4 | 26.9 | 29.2 | 36.2 | – | 79.5 | 100 | – | – | – | – | – | – |
|  | % | – | – | – | – | – | 0.6 | – | – | 0.6 | – | 1.2 | – | 4.7 | – | 9.9 | – | 5.8 | 0.6 | 3.5 | 2.3 | 7.0 | – | 43.3 | 21.1 | – | – | – | – | – | – |

–, not applicable; AMC, amoxicillin/clavulanic acid; AMP, ampicillin; AMX, amoxicillin; AZM, azithromycin; CDR, cefdinir; CEC, cefaclor; CFM, cefixime; CLR, clarithromycin; CPD, cefpodoxime; CRO, ceftriaxone; CTB, ceftibuten; CTX, cefotaxime; Cum., cumulative; CXM, cefuroxime; LVX, levofloxacin; MXF, moxifloxacin; SXT, trimethoprim/sulfamethoxazole; TET, tetracycline.

Bold vertical bars in table correspond to the CLSI-susceptible breakpoints.
